# Updated EBMT/ESID inborn errors working party guidelines for haematopoietic stem cell transplantation for inborn errors of immunity and metabolism

**DOI:** 10.1038/s41409-026-02906-0

**Published:** 2026-05-22

**Authors:** M. H. Albert, A. C. Lankester, R. Wynn, A. R. Gennery, M. Hoenig, A. Kreins, E. C. Morris, M. Slatter, M. E. Bernardo, B. Neven

**Affiliations:** 1 Department of Pediatrics, Dr. von Hauner Children’s Hospital, University Hospital, LMU, Munich, Germany; 2 Department of Pediatrics, Willem-Alexander Children’s Hospital, Leiden University Medical Center, Leiden, The Netherlands; 3 Blood and Marrow Transplant Programme, Royal Manchester Children’s Hospital, Manchester, UK; 4 Translational and Clinical Research Institute, Newcastle University, and Paediatric Immunology and Haematopoietic Stem Cell Transplantation, Great North Children’s Hospital, Newcastle upon Tyne, UK; 5 Department of Pediatrics, University Medical Center Ulm, Ulm, Germany; 6 Infection Immunity and Inflammation Research and Teaching Department, University College London Great Ormond Street Institute of Child Health, London, UK; 7 Department of Immunology and Gene Therapy, Great Ormond Street Hospital for Children NHS Foundation Trust, London, UK; 8 UCL Institute of Immunity and Transplantation, UCL, London, UK; 9 Department of Haematology, University College London Hospitals NHS Foundation Trust, London, UK; 10 Department of Immunology, Royal Free London Hospitals NHS Foundation Trust, London, UK; 11 Pediatric Immunohematology and Bone Marrow Transplantation, IRCCS San Raffaele Scientific Institute, Milan; San Raffaele Telethon Institute for Gene Therapy (SR-Tiget), Milan, ‘Vita-Salute’ San Raffaele University, Milan, Italy; 12 Pediatric Unit of Immunology, Hematology, and Rheumatology, Necker Hospital, Paris, France; 13 IMAGINE Institute U1163, Paris, France; Paris Cité University, Paris, France

**Keywords:** Translational research, Bone marrow transplantation

## Introduction

In 2021, the joint Inborn Errors Working Party (IEWP) of the European Society for Blood and Marrow Transplantation (EBMT) and the European Society for Immunodeficiencies (ESID) published guidelines for haematopoietic stem cell transplantation (HSCT) of inborn errors of immunity (IEI) [1]. This expert consensus statement has become a valuable clinical reference for physicians across the world. Significant advances in the field require an update to these guidelines, which now also cover inborn errors of metabolism (IEM), thereby better reflecting the scope of the IEWP. It builds on the previous manuscript, adding new recommendations where additional knowledge has been gained.

Currently, more than 500 monogenetic IEI are reported, disease-causing genetic variants are being identified in a growing number of patients and discoveries of genetic mechanisms, such as monoallelic expression, have made it easier to understand clinical phenomena such as incomplete penetrance [2, 3]. Recognition of a growing number of IEI resulting from somatic mutations in specific cell lineages has added an additional level of complexity. Treatment of IEI by HSCT is increasingly successful and the field has expanded from predominantly paediatric to include adult medicine [4]. The IEWP continues to contribute to this success by being a forum for translating scientific discovery into improved clinical care [4–11], sometimes published jointly with and complemented by findings of the North American Primary Immune Deficiency Treatment Consortium (PIDTC) [12–15].

The clinical heterogeneity of patients and reliance on observational rather than prospective studies currently preclude strictly defined transplant protocols for IEI patients. These guidelines include several protocol options based on published data, centre experience and expert opinion. Whenever possible, transplant procedures should follow these recommendations, but modifications may be needed according to the respective IEI variant and patient condition. The IEWP continues to recommend that all IEI patients be transplanted in experienced centres that regularly treat such patients and participate in the IEWP in order to ensure continuous outcome improvement. Centres are strongly advised to register their transplanted patients in the EBMT and ESID registries, which will allow continuous evaluation of the outcomes in patients treated in line with IEWP guidelines [16].

For acutely life-threatening IEI the decision for HSCT is clear, but for many others it is less straightforward and requires careful consideration of advantages and disadvantages. The decision process must weigh multiple factors, including the best possible preparation of a patient/family for HSCT. New knowledge was gained on the outcome of specific IEI and on treatment with targeted therapies in recent years [6]. This updated guideline contains a dedicated paragraph on such pre-HSCT considerations.

Autologous stem cell gene therapy (GT)—which is not the primary focus of these guidelines—has been clinically tested for a limited number of IEI and IEM. One GT product for IEI (Strimvelis® for ADA-Severe Combined Immunodeficiency, SCID) has obtained market authorisation by the European Medicines Agency [17]. Recently, the largest study in an IEI so far with lentiviral-based GT has been reported in ADA-SCID [18]. For patients with IEM, two lentiviral GT products are available on the European (Libmeldy® for MLD) and US (Lenmeldy® for MLD and Skysona® for X-ALD) markets [19]. GT offers the advantage of avoiding negative consequences of alloreactivity (GVHD), but concerns remain about the curative potential of a mixed chimeric state in certain non-SCID IEI, which is inherent to current GT approaches and the possible risk for insertional mutagenesis. However, in the absence of comparative studies, it is impossible to make firm recommendations on the hierarchical position of GT in comparison to HSCT. It has to be considered that: (a) long-term safety and efficacy data of GT are still limited, and (b) comparing outcome data from prospective single- or oligo-centre studies (as is the case for GT) with retrospective multi-centre studies (as most of the evidence for HSCT) does not meet evidence-based scientific standards. For patients with a particular IEI, participation in a GT study may be considered for patients lacking a matched (family) donor and able to access a study centre.

## Pre-transplant considerations

### Indications for HSCT in IEI

Allogeneic HSCT offers a curative option for many IEI, provided that the defective gene is expressed in haematopoietic-derived cells. However, indications and optimal timing for HSCT vary greatly depending on the specific condition.

To support clinical decision-making, the EBMT classifies transplant indications into four categories based on available evidence and risk-benefit assessment: standard of care, clinical option, developmental and generally not recommended, which we have refined here, taking into account that in this field of very rare diseases, any indications guideline will never be completely non-ambiguous and up to date [20].

In diseases where HSCT is considered standard of care, the decision to proceed is typically straightforward, including with alternative donors. SCID falls in this category since the disease leads to early mortality unless treated by HSCT. Similarly, familial hemophagocytic lymphohistiocytosis (HLH) is a clear indication for HSCT, as soon as remission of hyperinflammation is reached to optimize survival. In most genetic forms, pre-emptive transplantation has to be considered. Leukocyte adhesion deficiency, certain combined immunodeficiencies such as MHC class II or diseases with immune dysregulation, such as IL10R deficiency, also fall into this category. In diseases such as Wiskott-Aldrich syndrome or chronic granulomatous disease (CGD), growing international experience and retrospective data have helped define the long-term natural history and HSCT outcomes [5, 21–23]. This supports HSCT as standard of care, even as a pre-emptive measure in many patients with these conditions and facilitates informed counselling and decision-making.

The EBMT classifies the indication for HSCT for the large group of combined immunodeficiencies (CID) and other IEI as standard of care when matched donors are available. However, ‘clinical option’ may sometimes be the more adequate category, as the final decision is more complex and must integrate multiple factors: the natural history of the disease, genotype/phenotype correlation if known, quality of life, disease burden and associated comorbidities and age of the recipient. Transplant-related considerations include donor availability and characteristics, short- and long-term risks (including fertility) and availability of alternative therapies. Lastly, the motivation and understanding of the patient and their family must be carefully assessed and supported. Pre-transplant comorbidities are increasingly recognized as major predictors of post-HSCT outcomes, emphasizing the need for timely intervention before irreversible damage or treatment-related complications occur [4, 24]. Such nuanced decisions benefit from multidisciplinary discussions involving teams with expertise in both IEI and transplantation. The aim is to define the optimal therapeutic strategy, tailored to the patient’s disease manifestations and expected clinical course. In Table 1a we summarize these categories of indications.

**Table 1:** **a** EBMT indication categories for HSCT in patients with IEI. **b** EBMT indication categories for HSCT in patients with IEM and osteopetrosis.

a				
	standard of care^a^	clinical option^a^	developmental^a^	generally not recommended^a^
**indication for HSCT**	Indication for timely and preemptive HSCT in all patients because of early mortality unless treated by HSCT	Indication for HSCT (including preemptive) is complex and depends on multiple factors, including natural history of the disease, comorbidities, age, willingness, individual disease course, donor availability, etc.	Indication for HSCT may be made if there is a sufficient rationale and expectation that a patient will benefit from it, but it is often based on single case reports	generally not an indication for HSCT because the defect is either extrahematopoietic or if some extrahematopoietic disease manifestations (e.g. neurologic) are known to progress despite HSCT
**diseases**	• SCID (all patients) • familial HLH • IL-10 receptor deficiency • WAS (many patients) • CGD (many patients)	• all IEI demonstrated to be curable by HSCT	• IEI where experience is limited to isolated case reports, like some PIRD (e.g. haploinsufficiency of A20) • IEI where incomplete disease correction is expected because of extra-hematopoietic manifestations (e.g. STAT3-LOF)	• thymic epithelial defects • i.e. AT • HSCT may still be indicated in select patients, where a positive risk/benefit ratio is expected

b				
	standard of care	clinical option	developmental	generally not recommended
**Indication for HSCT**	Indication for timely HSCT in all patients because of the progressive nature of the disease	Indication for HSCT depends on multiple factors, including natural history of the disease, failure to standard of care, comorbidities, age, willingness, individual disease course, donor availability, etc.	Indication for HSCT may be made if there is a sufficient rationale and expectation that a patient will benefit from it	generally not an indication for HSCT because disease manifestations are known to progress despite HSCT
**diseases**	• MPSI • MPSII • alpha-mannosidosis • X-ALD with early cerebral involvement • osteopetrosis with *TCIRG1*, *RANK*, *CLCN7* (unless neuropathic form) variants or genetically undefined forms	• MPSVI (if ERT failed or is not possible) • MLD (only early and/or asymptomatic); for late infantile and early juvenile, GT is the preferred option • Krabbe (early infantile, pre-symptomatic) • Aspartylglucosaminuria • Fucosidosis • MNGIE • Gaucher • Wolman	• There are many rare LSD in which the efficacy of HSCT is not established. Consider a discussion with the expert centre • HSCT might be considered in individual cases where the child is in good clinical condition	• osteopetrosis with *OSTM1*, *RANKL* variants and patients with neuropathic *CLCN7* • MPSIIIA

^a^Given the rarity of some IEI, variable presentations and other factors outlined in the manuscript text, none of these categories can be interpreted as sharply distinct. Almost any IEI may fall into any of the categories, depending on patient and disease factors.

While transplant-related comorbidities weigh heavily on outcomes, recent advances in HLA-mismatched transplantation have expanded access to HSCT in patients lacking an HLA-matched donor [9]. This may shift the risk-benefit balance towards earlier transplantation, even in conditions where historically, a watch-and-wait approach was preferred in the absence of a matched donor.

Importantly, many IEI are ultra-rare and published natural outcome experiences remain highly variable or limited. This highlights the importance of both natural history studies and HSCT outcome registries, which help refine indications and identify optimal timing [10, 24–28].

For many other IEI, experience is limited to isolated case reports. These conditions, including many primary immune regulatory disorders (PIRD), generally fall within the ‘developmental’ category. PIRD are characterized by a broad spectrum of autoimmune and autoinflammatory manifestations. In these diseases, inflammation should be controlled as much as possible before considering HSCT. The role of emerging biotherapies and targeted immunosuppressants remains to be clarified, either as definitive treatment or as a bridge to transplantation.

Monogenic autoinflammatory diseases (e.g. DADA2 [29]) represent a distinct and expanding subgroup of IEI and have emerged as potential candidates for HSCT, although current experience remains limited and indications are still considered developmental. Somatic gain-of-function mutations (e.g. TLR8-GOF [30]) raise concerns about post-transplant disease recurrence in the setting of mixed chimerism or residual mutant clones. In addition, when the disease-causing gene is also expressed outside the haematopoietic compartment, some clinical features may not be corrected by HSCT.

### Comorbidities

Patients with IEI are frequently exposed to several factors predisposing them to the development of comorbidities prior to HSCT. Infections play a central role, both through direct clinical consequences and indirect organ damage. The nature and frequency of infections are highly dependent on the underlying immune defect. Active infections at the time of transplantation are associated with inferior outcomes in multiple settings [7, 8, 10]. Specific pathogens can be particularly deleterious. Mycobacterial infections in NEMO deficiency or MSMD or chronic cryptosporidiosis in CD40L deficiency and other CID are such examples [24]. Profound immunosuppression peri-transplant may exacerbate pre-existing infections. It is strongly recommended to treat all active infections prior to HSCT when possible and identify any antimicrobial resistance. In certain cases, especially with viral pathogens, only immune reconstitution via HSCT may allow for definitive infection control. Asymptomatic carriership of pathogens such as BCG, adenovirus in the gastrointestinal tract, or persistent respiratory viruses in the respiratory tract can become clinically significant post-transplant.

Permanent organ damage represents a major barrier to successful HSCT in patients with IEI [4, 10, 24]. Lung and liver involvement are frequent in IEI and can severely impact transplant outcomes. Pulmonary damage is often heterogeneous, a consequence of infection and/or inflammation, while renal impairment may also result from repeated nephrotoxic treatments. Bronchiectasis is particularly prevalent in older patients. In patients with severe, permanent liver or lung injury, treosulfan-based regimens may be preferred to minimize the risk of veno-occlusive disease (VOD) and pulmonary toxicity [31]. Several attempts have been made to quantify pre-HSCT comorbidity burden, including the HSCT-specific comorbidity index (HSCT-CI) and, more recently, the IDDA score [32, 33]. Both scoring systems have been shown to predict outcome following HSCT, although the latter remains under evaluation and may better capture the specific complications in IEI patients.

### Bridging (including targeted) therapies

Increasingly, the focus of pre-transplant optimization lies in the control or mitigation of immune dysregulation. Historically, this has often been attempted (and sometimes achieved) with steroids, with all their known deleterious off-target effects.

A paradigmatic example is HLH, in which early studies have demonstrated that achieving remission of the hyperinflammatory state prior to conditioning is essential to ensure engraftment and reduce transplant-related mortality [34]. Among the key mediators of this hyperinflammatory state, interferon-gamma (IFN-γ) has been identified as a major obstacle to successful engraftment. Its deleterious effects on the haematopoietic niche and immune environment have been demonstrated in both clinical and experimental settings [35]. Targeted inhibition of IFN-γ with emapalumab or JAK inhibitors offers promise to improve engraftment success and reduce transplant-related toxicity, not only in HLH but also in other interferon-driven PIRD [36].

Targeted therapies have shown efficacy when administered pre-transplant, like abatacept in patients with CTLA4 or LRBA deficiencies or leniolisib in activated PI3Kδ syndrome (APDS), helping stabilize autoimmune and lymphoproliferative features [11, 37–40]. In still anecdotal yet illustrative cases, anti-IL-18 therapy may play a role in IL-18-driven conditions [41]. These examples highlight the broader principle that effective control of inflammation prior to transplant is critical, especially in PIRD, even though complete remission of inflammatory manifestations cannot always be achieved and should ultimately not delay HSCT.

### HLA-typing

High resolution, allele level HLA-A, -B, -C and -DRB1, -DQB1 typing is mandatory for all donors except unrelated cord blood (CB), with DP becoming increasingly relevant. For unrelated donors, allele-level matching at all 10 loci is the current gold standard. Unlike in malignant diseases, 9/10 mismatched unrelated donors have not been demonstrated to be equivalent in inborn errors, which is why many centres prefer to apply T-cell depletion approaches, as recommended below.

### Donor hierarchy

In the last decade, HSCT outcomes in IEI have steadily improved for the different donor types due to improved HLA typing technology and donor selection, better management of HSCT complications, and improved supportive care. Survival rates obtained with matched unrelated donors are now similar to those of matched sibling donors [5, 22, 24, 42]. The higher rates of HSCT complications and often higher donor age with matched unrelated donors still justify the position of HLA identical siblings as the first choice in most cases. That notwithstanding, family donor screening should check whether a candidate donor is affected by or a carrier of the same genetic defect, especially in diseases with a late or variable onset of clinical presentation [43, 44]. In IEM, a carrier sibling should generally be excluded as a donor since he/she would provide lower than normal levels of enzyme. If a higher stem cell dose is felt to be required, it may not be possible to obtain peripheral blood stem cells (PBSC) from an underage sibling unless they are able to consent to the administration of G-CSF.

### Recommendations for haploidentical HSCT and mismatched unrelated donors

In recent years, HSCT performed with mismatched family and unrelated donors has demonstrated improved and encouraging results [8, 9, 45] and can be considered a reasonable alternative in the absence of a matched donor. However, the higher complexity of these transplants supports that they should be performed in centres with experience in these procedures.

Based on published data and the experience from participating IEWP centres, two recommendations are made for transplantation with MMFD and MMUD based on the TCRα/β depletion approach and the T-replete marrow PT-Cy approach in Table 2 [9, 46–50]. In terms of which to use, a recent large retrospective IEWP study found that both have specific advantages and disadvantages, but that pre-HSCT comorbidity was the most important determinant of outcome, not the approach used [8]. PT-Cy is cheap, readily available and easy to administer, but was associated with higher rates of toxicity and acute GvHD in the IEWP study. TCRα/β depletion is more expensive and requires specialist laboratory expertise and was associated with higher rates of adenoviraemia, primary graft failure and requirement for second HSCT [8]. CD34⁺ positive selection is no longer routinely recommended, but may remain an acceptable option in selected SCID patients, when other approaches are not feasible or available.

**Table 2:** Recommended platforms for HLA-haploidentical HSCT.

	TCR α/β [130–132]	PT-Cy [47, 48]
**Protocols**	A, B, C, D	A, B, C, D
**Graft**	TCR α/β - CD19 depleted PBSC	unmanipulated BM (1st choice) or PBSC^a^ (2nd choice)
**Cell dose**	10–20 × 10e^6^ CD34/kg	3–5 × 10e^8^ TNC/kg
**Serotherapy**	ATLG/Grafalon®: 3 × 4 mg/kg (d −4 to −2) Rituximab: 200 mg/m^2^ (d-1) *If ATLG is not available:* ATG/Thymoglobuline® 3 x 3 mg/kg (d-12 to −10)^b^	Alemtuzumab: 2 × 0.2 mg/kg (d-10 to −9) *If Alemtuzumab is not available:* ATG/Thymoglobuline® 3 × 2.5 mg/kg (d-10 to −8)^c^
**GVHD prophylaxis**	if αβ T cells in graft ≥ 10e5/kg: add CSA	cyclophosphamide 50 mg/kg on d + 3 and d + 4 tacrolimus or CSA from d + 5 until at least d + 100 MMF from d + 5 to d + 35

^a^ In case PBSC are used, higher rates of cGVHD can be expected and additional or prolonged GvHD prophylaxis may be considered [133].

^b^ Consider to lower dose in adolescents and young adults with low lymphocyte counts and to increase the dose in young children with high lymphocyte counts [134].

^c^ If using ATG in PT-Cy protocol, additional rituximab may be considered.

### Stem cell source and dose

Concerning the stem cell source, most centres would prefer bone marrow (BM) for unmanipulated grafts but would prefer PBSC in case in vitro graft manipulations are needed.

In case of unmanipulated BM grafts in matched family or unrelated donors, the recommended cell dose is 2–4 × 10^8^ TNC/kg. In case of T-replete PBSC grafts from matched donors, the recommended dose is 5–8 × 10^6^ CD34 + /kg. For mMUD/MMFD, specific recommendations are provided in Table 2. Higher stem cell and T-cell content of the graft—as achieved with PBSC—may help overcome engraftment barriers with reduced intensity regimens [51], but caution is advised with very high T-cell doses ( ≤ 5 × 10^8^/kg) [52].

In recent years, the number of unrelated CB transplants has decreased due to new methods used for haploidentical transplants. In some centres, cords remain the preferred stem cell source when an alternative donor is required. Unit selection is based on allele-level HLA-A, -B, -C and -DRB1 matching and cell doses. All units should be ≥7/8 matched to the recipient. Historically, priority is given to an 8/8 matched single unit CB with a TNC ≥ 3.0 × 10e^7^/kg, followed by a 7/8 matched single unit CB with a TNC ≥ 5.0 × 10e7/kg. CD34+ counts are not standardised but recommended >1.7 × 10e^5^/kg [53], although the optimal dose criteria remain to be defined [54].

### Conditioning and serotherapy

#### General considerations

Conditioning refers to the preparative regimen used to eliminate affected autologous immuno-haematopoiesis and create space for donor haematopoietic stem and progenitor cells in the BM and thymic niches. Restraint in the use of (full) conditioning is recommended for very young and premature infants (age <8wk), patients with major comorbidities and patients in whom a first-step unconditioned infusion of an allograft may be life-saving in the short term, particularly in SCID patients with active infections (see section on SCID).

Chemotherapy regimens in these guidelines combine agents with primarily myeloablative (busulfan, treosulfan, thiotepa and melphalan) and immune-ablative potential (fludarabine and cyclophosphamide). Next to chemotherapy, antibody-based immunotherapy (serotherapy), including rituximab, anti-thymocyte globulin (ATG), anti-T lymphocyte globulin (ATLG) and alemtuzumab, is used to eliminate host immunity, aiming to prevent rejection and reduce the risk for GvHD. Uniform dosing strategies for the conditioning agents based on body weight or body surface area have been increasingly replaced by more individualized approaches, including therapeutic drug monitoring (TDM) and model-informed precision dosing (MIPD) [55]. The value of these approaches to improve efficacy and limit toxicity was first demonstrated for busulfan, for which it is currently considered the standard of care [56]. In recent years, evidence for the added value of TDM and MIPD to optimize use of other agents is accumulating [55, 57–61]. However, these studies are still limited, emphasizing the need for additional studies. There is evidence to suggest that younger patients require higher exposure to busulfan and melphalan in order to achieve full donor chimerism, although this has not led to specific age-dependent dosing recommendations [62, 63]. Similar to chemotherapy, the impact of serotherapy exposure on clinical outcome parameters, including T-cell reconstitution, overall and event-free survival, has been demonstrated in a number of clinical studies [64–66]. While this has already led to altered treatment regimens, involving both dosing and timing in relation to infusion of the graft and the availability of online tools for MIPD, formal prospective controlled studies are awaited [57]. Of note, most of these studies have been performed in non-IEI/IEM or mixed cohorts, including both malignant and non-malignant diseases and mainly in HSCT with T-replete grafts. The regimens recommended here have been successfully used by large European expert centres and others. Nevertheless, other regimens may be suitable as well for HSCT in inborn errors. For example, a reduced intensity combination of fludarabine, melphalan and thiotepa was found to be safe and efficacious in patients with HLH and patients >1 year of age with other IEI [67, 68].

#### Busulfan

**Fig. 1:**
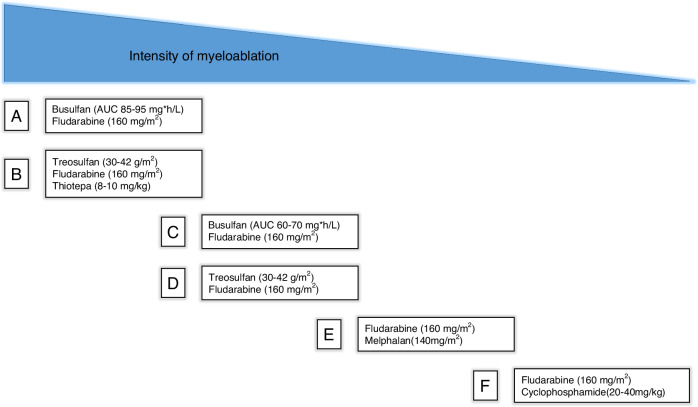
Conditioning regimens. Protocol **A** and **B**: Recommended for patients without severe pre-existing organ damage and non-SCID diseases where a complete donor chimerism is desired for optimal disease correction. Protocols **C** and **D**: Recommended for patients with pre-existing organ damage, older age and/or diseases where engraftment has been shown to reliably occur with reduced toxicity conditioning. Mixed donor chimerism is more likely to occur compared to protocols **A** and **B**. Protocol **E**: This may be suitable for patients with pre-existing organ damage and/or diseases where full myeloid engraftment is not absolutely required. Higher degrees of chimerism can be achieved when using PBSC. Protocol **F**: This regimen is only recommended for DNA repair/radio-sensitivity disorders (except Artemis deficiency) in which alkylating agents are used at a low dose.

Therapeutic drug monitoring (TDM) is mandatory. Busulfan may be administered in one to four daily doses as per institutional standard. The initial busulfan dose is based on weight or body surface area, according to either the manufacturer-recommended nomogram or the one published by Bartelink et al. [69], while subsequent doses are based on TDM [70, 71]. Repeated TDM is recommended in case the first dose adjustment is >25% and in infants [72, 73]. EBMT recommendations for busulfan TDM have been published [56]. Here, two busulfan regimens are recommended: a myeloablative protocol A (85–95 mg*hr/L) and a reduced intensity protocol C (60–70 mg*hr/L; Fig. 1 and Table 3). In general, higher busulfan exposure results in a higher likelihood of complete donor engraftment [71] and a recent study found that children <3 years may benefit from a higher exposure ( ≥ 75 mg*hr/L), taking into account the risk for VOD [62].

**Table 3:** Chemotherapy protocols A-F.

	days to HSCT	−6	−5	−4	−3	−2	−1	0
protocol								
**A**								
Busulfan (AUC 85–95)		x	x	x	x			
Fludarabine (4 × 40 mg/m2)^a^		x	x	x	x			
**B**								
Treosulfan (3 × 10–14 g/m2)^b^			x	x	x			
Fludarabine (4 × 40 mg/m2)		x	x	x	x			
Thiotepa (1 × 8/2 × 5 mg/kg)		x						
**C**								
Busulfan (AUC 60–70)		x	x	x	(x)			
Fludarabine (4 × 40 mg/m2)		x	x	x	x			
**D**								
Treosulfan (3 × 10–14 g/m2)^b^			x	x	x			
Fludarabine (4 × 40 mg/m2)		x	x	x	x			
**E**								
Melphalan (1 × 140/2 × 70 mg/m2)						x		
Fludarabine (4 × 40 mg/m2)		x	x	x	x			
**F**								
Cyclophosphamide (4 × 5 mg/kg)			x	x	x	x		
Fludarabine (4 × 40 mg/m2)		x	x	x	x			

^a^In infants <12 months of age, body weight-based dosing using the 30-rule may be used [83]: 1,33 mg/kg instead of 40 mg/m².

^b^3 × 10 g/m^2^ in < 0.4m^2^ body surface area, 3 × 12 g/m^2^ in 0.4 – <0.9 m^2^, 3 × 14 g/m^2^ in ≥ 0.9 m^2^.

Serotherapy is recommended in all alternative donor transplants and may be considered when using HLA-matched siblings (see paragraph ‘ATG/ATLG and alemtuzumab’). The recommended dose ranges for unmanipulated HLA-matched grafts are: ATG/Thymoglobulin® 5–10 mg/kg (total dose, usually split over 2–3 days), ATG/Grafalon® 15–30 mg/kg (total dose, usually split over 3 days) and alemtuzumab 0.6–1.0 mg/kg (total dose, administered at 0.2 mg/kg/day). Specific recommendations are provided for the two haplo-protocols (Table 2).

#### Treosulfan

Treosulfan is known for both its myeloablative and immune suppressive activity and its reported favourable toxicity profile [31, 74–78]. Similar overall survival and outcome have been reported with myeloablative busulfan and treosulfan-based regimens in malignant and non-malignant diseases [22]. Still, the predictability and potential to reach full donor chimerism are less in current treosulfan regimens compared to high AUC targeted busulfan regimens, which may be relevant in those diseases where full donor chimerism is preferred [5, 78]. While treosulfan exposure has been linked to clinical outcome parameters, pharmacological studies have not yet provided convincing support for routine use of TDM [60, 79, 80]. Current treosulfan dose recommendations are based on body surface area (Table 3) [81], while some centres are applying TDM/MIPD [82]. In these guidelines, two treosulfan-based regimens are proposed, B (myeloablative) and D (reduced intensity), with additional thiotepa in regimen B (Fig. 1).

#### Fludarabine

Fludarabine is primarily a lymphodepleting agent. Recommendations on dosing are based on body surface area, preferably making use of a four-day regimen [57]. In infants, Fludarabine dosing may be more accurate on a body weight basis [83]. MIPD seems to be a useful tool to limit interindividual variability in exposure [58] and seems to result in improved outcomes, but these results require confirmation in larger studies [57, 84].

#### Cyclophosphamide

Whereas cyclophosphamide has been largely replaced by fludarabine because of a more favourable risk/benefit profile, it is still recommended at a significantly reduced dose and combined with fludarabine in protocols used for patients with Fanconi anaemia and other DNA-repair disorders [85]. In recent years, cyclophosphamide has been increasingly used post-transplant (PT-Cy) as an in vivo T-cell depletion strategy, particularly in mismatched donor transplantation, while the role of TDM is unclear [86].

#### Thiotepa

Thiotepa is frequently used with the aim of increasing the myeloablative potential of conditioning regimens, particularly in regimen C. Moreover, thiotepa has excellent central nervous system penetration, which may be beneficial in specific diseases but may also increase the risk for toxicity, especially for transplant-associated thrombotic microangiopathy [87]. Whether thiotepa TDM has added value is the subject of ongoing studies.

#### Melphalan

Originating from a time when historical use of busulfan and cyclophosphamide was associated with significant toxicity in patients with pre-existing end-organ damage, less myeloablative regimens with fludarabine and melphalan were regularly used (protocol E) [88]. While results were encouraging, mixed chimerism was common and toxicities in infants remained significant [51]. Recent reports suggest that TDM—also taking renal impairment into account—may optimize exposure and clinical outcome [89, 90]. Still, with current busulfan- and treosulfan-based reduced toxicity regimens, the need for regimen E is limited.

#### ATG/ATLG and alemtuzumab

T-cell-depleting serotherapy is considered standard in all unrelated and mismatched family donor transplants. It is also increasingly used in HLA-identical family donor transplants, particularly in diseases with an inflammatory component [91]. Different biological products may be used to achieve these goals: polyclonal rabbit ATG (Thymoglobuline®) and ATLG (Grafalon®) and anti-CD52 monoclonal antibody alemtuzumab. Recent studies in mixed populations of diseases have indicated that ATG and alemtuzumab exposure following standard dosing is highly variable, with an impact on clinical outcome [64–66]. Moreover, body weight and lymphocyte numbers at the start of serotherapy treatment have been reported as important parameters [65, 92, 93]. Although probably equally important in the heterogenous population of patients with IEI, it remains difficult to provide a specific and individualized recommendation on dose and timing, in particular based on current knowledge and published data [94]. PK/PD studies are ongoing that may eventually result in more specific and individualized recommendations [66]. Current recommendations are made based on available, albeit limited, published data and common practice in experienced centres. The recommended dose ranges for unmanipulated, HLA-matched grafts are given in Table 3. To avoid overexposure of serotherapy resulting in prolonged lympho-/immunodepleting activity following infusion of the graft, serotherapy administration may be scheduled more distal to the graft, i.e. starting on day -10/-8. The latter is particularly important in lymphopenic individuals and when using cord blood grafts [94, 95]. Based on published data, specific recommendations on serotherapy are provided for haplo-identical/mismatched donor HSCT in the context of the TCR α/β depletion approach and the T-replete marrow PT-Cy approach (Table 2). Making use of available online MIPD tools or consulting centres with TDM/MIPD experience may be considered for more individualized dosing to further optimize clinical outcomes.

### Graft versus host prophylaxis

We continue to recommend GvHD prophylaxis with a calcineurin inhibitor plus a second agent (MTX or MMF) for unmanipulated grafts. Evidence for the routine use of potentially improved regimens incorporating substances like abatacept or vedolizumab is not yet sufficient. If unmanipulated PBSC from matched unrelated donors are used, limiting T-cell content of the graft or prolonged GvHD prophylaxis may be considered [52]. In mismatched family and mismatched unrelated donors, graft manipulation (either ex vivo or in vivo) should be strongly considered, depending on the degree of mismatch, to limit the risk for GvHD, using the TCR α/β depletion or PT-Cy approach, respectively [8].

## Specific recommendations

### Severe combined immunodeficiency

#### Diagnostic and prophylactic considerations

SCID is a heterogeneous group of inherited disorders with impaired T-cell differentiation and function. Beyond the detection of a variant in a SCID-associated gene, the revised PIDTC criteria can be helpful to differentiate between SCID and CID [96]. The presence of B- or NK-cells depends on the genetic defect and its impact on the differentiation of these lineages. Historically, it was used for the classical immunophenotypical nomenclature.

Profound knowledge about the immunological background of these disorders is crucial for diagnosis and the evolution of patient-tailored therapeutic strategies. Management in experienced and dedicated centres is therefore highly recommended. While there is a large variability across centres, we have tried to make consensus suggestions for clinical implications of confirmed severe T-cell lymphopenia detected by NBS in Table 4.

**Table 4:** Suggested management of SCID patients newly diagnosed by NBS or family history.

action recommended with abnormal TREC-screening result (and confirmation by flow cytometry) or clinical diagnosis of SCID	rationale
stop breastfeeding immediately until the CMV status of the mother is determined	avoid CMV infection before HSCT
continue to stop breastfeeding or pasteurise milk if the mother is CMV-seropositive	avoid CMV infection before HSCT
isolate the patient according to local and individual situation and circumstances	avoid community acquired (predominantly viral) infections before HSCT
plan HSCT to be performed before the age of 3 months	shorter period at risk for infections
live vaccines are strictly contraindicated	avoid vaccine strain infections
irradiate blood products	avoid transfusion-related GvHD
initiate PJP prophylaxis	avoid PJP
start Ig replacement therapy	passive transfer of humoral immunity
treat with two tuberculostatic drugs^a^ in infants vaccinated with BCG	prevent BCGitis and Infection-associated Immune Reconstitution Inflammatory Syndrome (IRIS)
treat with four tuberculostatic drugs^a^ in infants with BCGitis	control BCGitis

^a^Tuberculostatic drugs should be paused during conditioning as drug interactions and potentially serious toxic side effects need to be considered.

Defining a genetic diagnosis before transplantation is highly desirable, especially in patients detected by NBS. However, urgent clinical decisions must not be delayed. We strongly recommend a parallel approach of preparation for definitive treatment (donor search) and expedited genetic diagnostics.

If not detected via newborn screening (NBS) or by family history, patients with SCID present with serious, persistent and potentially life-threatening viral, fungal, bacterial and opportunistic infections within the first year of life. However, not all SCID patients who are at risk for such a presentation will be detected by NBS based on the determination of TREC levels, and not all patients with ‘typical’ opportunistic and life-threatening infections will fulfill the criteria of SCID. SCID related to radiosensitivity disorders will be discussed below.

Maternal T-cells can cause GVHD-like symptoms (exanthema, diarrhoea, liver disease) and potential organ damage in SCID patients. Patients with typically hypomorphic mutations (with residual protein-expression and function) can develop a clinically and immunologically heterogeneous phenotype. This includes: Omenn’s syndrome (defined by generalized erythema, polyadenopathy, hepatosplenomegaly, eosinophilia, alopecia and oligoclonal expansion of autoreactive T cells), or autoimmunity and granuloma, or CID with higher numbers of autologous lymphocytes [97]. Both maternal engraftment and Omenn’s require early administration of immunosuppressive drugs, sometimes including serotherapy.

**Fig. 2:**
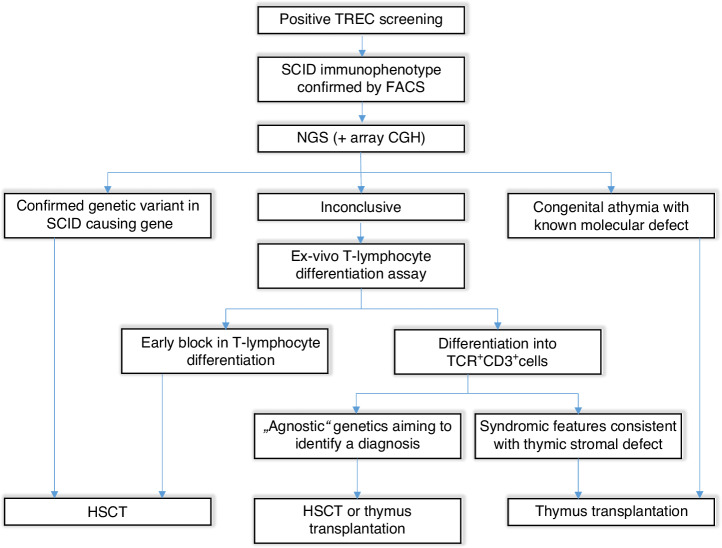
Diagnostic and therapeutic pathway for a child with a positive TREC newborn screening. Adapted from Golwala et al., Clin Immunol 2025129 [135]. In genetically undiagnosed patients, in particular with isolated T-cell lymphopenia, ex vivo T-cell differentiation assays can help distinguish haematopoietic-intrinsic defects requiring HSCT from thymic stromal defects requiring thymus transplantation. Despite successful ex vivo differentiation of TCR + CD3+ cells, further agnostic genetic testing is warranted in absence of syndromic features suggestive of a thymic stromal cell defect. CGH comparative genomic hybridization, NGS next-generation sequencing, TCR T-cell receptor, TREC T-cell receptor excision circles.

In patients with a T-B+NK+ immunophenotype, there is a risk of performing unnecessary and ineffective HSCT if the selective T-cell defect is not caused by a primary haematopoietic defect but by the absence of thymic function. Congenital athymia is a life-limiting disorder requiring corrective treatment with allogeneic thymus transplantation if accessible [98]. In athymic patients, definitive reconstitution of immune function will not be achieved by HSCT, and survival post-HSCT is poor; it may, however, be indicated in infants with severe viremia, especially if an MSD/MFD is available [98]. Thymic stromal defects can be associated with syndromic features, but these can be mild or even absent. Thus, for a patient with a genetically undefined T-B + NK+ immunophenotype, we recommend actively excluding congenital athymia by testing the intrinsic capacity of recipient haematopoietic stem cells to differentiate into T-cells ex vivo within an artificial thymic organoid model before proceeding to transplant [98]. Figure 2 suggests a potential algorithm for clinical decision-making in patients identified by TREC-based NBS.

For patients with ADA-SCID, three therapeutic options are available, including enzyme replacement therapy, GT and HSCT. For detailed information on how and when to use either of these, we refer to a recent review [99].

#### HSCT considerations

Conditioning is recommended as a default approach for SCID patients and is mandatory in Omenn’s syndrome. It increases the likelihood of myeloid engraftment, thymic output and independence from immunoglobulin substitution [7, 100]. The ‘ideal’ intensity is still a matter of debate and is currently being studied in a prospective trial (NCT03619551). We recommend protocols C or D for most patients. As there is limited experience in newborns with regard to pharmacokinetics, toxicity and tolerance of drugs used for conditioning, conditioned HSCT is not recommended before 6–8 weeks of age [101]. If a patient is not deemed fit to tolerate conditioning, an unconditioned rescue infusion may be performed, with a variable risk of absent B-cell reconstitution, of a decline in thymopoiesis over time, and high risk of graft failure in T-B-NK + SCID. An unconditioned infusion is associated with the expectation (and counselling of the family) that the patient will have to undergo a second, conditioned procedure when they have recovered without evidence of durable immune reconstitution. For patients transplanted without chemo-conditioning from MUDs, serotherapy is highly recommended to reduce the risk for GvHD [102].

### Radiosensitivity disorders

Patients with radiosensitive disorders such as DNA ligase 4 deficiency, Cernunnos-XLF deficiency, Nijmegen breakage syndrome or Ataxia telangiectasia (AT) may be detected by NBS or present with CID, autoimmunity, BM failure, myelodysplasia or malignancy, particularly leukaemia or lymphoma. Some may require transplantation [103]. There are too few data to recommend HSCT as the standard of care for most patients, although it should be considered for those detected on NBS. Conventional doses of alkylating agents are generally poorly tolerated, often leading to multi-organ failure and early transplant-related mortality. Best survival is achieved when patients receive a modified Fanconi-type conditioning regimen [85]. We recommend protocol F, but similar ones have been used successfully. Radiotherapy should be avoided due to catastrophic toxicity [104]. The long-term outcome of these patients following HSCT has yet to be determined, and the indication for HSCT should be set only on a case-by-case basis after careful consideration of risks and benefits. Careful long-term follow-up is recommended, particularly regarding secondary malignancies.

There are a few reports of HSCT for patients with AT. The same issues regarding sensitivity to radiation and alkylating agents apply to these patients. However, given the progressive neurological deterioration that AT patients experience, there is currently no place for routine HSCT for these patients but it may be discussed for selected individuals. The majority of those being considered for HSCT will have lympho-haematological malignancy requiring chemotherapy. Treatment schemes for malignancies need to be carefully adapted, also considering immunotherapy-based options whenever possible [105]. The optimal management of those picked up by NBS is yet to be determined, although they may improve immunologically over time without the need for HSCT.

### Adolescent and young adult (AYA) population

HSCT for adolescent and adult patients ( > 15years and into the seventh decade of life) has become increasingly more common in recent years, likely due to advances in genetic diagnostics, improved survival into adulthood with conservative treatment and recent data demonstrating good outcomes following HSCT in older IEI patients [4, 32, 106]. Complications tend to accumulate with age, which can result in end-organ damage, reduced quality of life and early death. However, identifying patients who may benefit from HSCT remains challenging, in part due to phenotypic heterogeneity and the absence of a genetic diagnosis in some [106]. Patients with higher HSCT-CI scores, active immune dysregulation or permanent organ damage have a worse outcome, while recipient age or donor type do not independently influence survival [4, 32], arguing for a more pre-emptive approach to HSCT in patients who have ongoing or recurrent IEI-related complications. Patients with phagocyte disorders (e.g. CGD) fare better than those with CID or primary antibody deficiencies [5].

In older patients and those with higher HSCT-CI scores, reduced intensity conditioning regimens are preferred to limit excess toxicity (C, D or E) [32, 107], while for younger patients with less significant comorbidities, regimens B and A have been successfully used [32, 108]. Special attention should be placed on the higher risk of GVHD in this patient group compared to children. As discussed above, the best possible control of infections, autoimmunity and autoinflammation is recommended pre-HSCT. Measuring IDDA score may be helpful to identify patients with uncontrolled inflammation or immune dysregulation [32]. Pre-HSCT counselling taking into account aspects such as fertility, sexuality, social issues and the limitation of HSCT to correct irreversible organ damage is especially challenging and important in this patient group [109].

### Osteopetrosis

Allogeneic HSCT is the therapy of choice for patients with infantile osteopetrosis, but it may also be considered in (older) patients with intermediate forms [110, 111]. Contraindications in specific subtypes must be excluded: (a) osteoblast defects: *RANKL* and (b) neurodegenerative forms: all patients with *OSTM1* variants and about half of patients with *CLCN7* [111]. Developmental delay, failure to thrive and rather specific EEG changes are early signs of neurodegenerative disease [111].

The incidence of graft failure is about 20%, implying a choice of myeloablative conditioning. In a recent large retrospective IEWP analysis in 746 patients, regimens B and A resulted in comparable survival and graft failure rates, but busulfan was associated with a significantly higher risk of VOD [112].

VOD, pulmonary arterial hypertension (PAH) and hypercalcemia are of special concern in osteopetrosis patients. For haplo-identical transplants, the PT-Cy approach is often preferred, since this protocol leads to more robust engraftment [8, 47]. Because of the high risk of disease-specific side effects in osteopetrosis transplants, HSCT should be performed in experienced centres only, in particular when using haploidentical donors.

### Metabolic diseases

IEM comprise a large group of inherited monogenetic diseases impairing the function of metabolic enzymes or proteins. Clinical manifestations often depend on the accumulation of substrates and can be multisystemic, frequently involving the central nervous system, visceral organs, the skeletal system and the heart [113]. We have adapted the above-mentioned EBMT indication criteria for IEM in Table 1b.

Some IEM are disorders of lysosomal, peroxisomal or mitochondrial function. HSCT is the therapy of choice for some of these conditions (X-ALD, MPSIH). In the lysosomal storage disorders (LSD), healthy donor cells deliver the missing enzyme to residual enzyme-deficient host cells, including in the brain, through a mechanism known as cross-correction. Enzyme replacement therapy (ERT), when available, might be given as a bridge before HSCT [114, 115]. ERT might be particularly indicated in patients with severe somatic comorbidities, which would otherwise increase TRM, such as in Wolman or severe MPSI-related cardiomyopathy [116, 117].

In LSD, there is a relationship between the enzyme delivered through HSCT and clinical outcome. In some diseases, including MPSIIIA and infantile MLD, there is no response to conventional HSCT, likely in part because the graft delivers insufficient enzyme. In others, including MPSIH, some organs, including the bone, remain relatively refractory to transplant [114, 118].

In all IEM, the timing of transplant is critical. Since a transplant prevents disease progression rather than reversing established disease, HSCT of children with advanced disease is associated with both poor disease and transplant outcomes. Introduction of newborn screening, as for Krabbe disease in some states in the US, will likely improve long-term outcomes as patients are diagnosed earlier [119]. Transplant decisions require multi-disciplinary input and informed family discussion. For example, a transplant might not be offered to boys with X-ALD presenting with advanced cerebral inflammatory disease, as assessed by Loes score on MRI scan, as well as cumulative neurologic symptoms.

The relationship between the amount of enzyme delivered and outcome has led to GT trials with the aim to improve enzyme delivery and outcome in LSD, including MLD, MPSI, MPSIIIA and MPSII. Such gene-addition GT approaches deliver supranormal enzyme doses and are likely safer than HSCT [120].

Regimen A is preferred since myeloablative conditioning is necessary to achieve full engraftment. Moreover, busulfan is employed in the neuropathic IEM with the aim of depleting resident microglial cells, which will be replaced by donor haematopoietic cells after their migration across the blood-brain barrier [121]. Occasionally, reduced-toxicity conditioning (regimen B) might be employed in somatic IEM such as Wolman or attenuated MPS. Serotherapy is recommended in both MSD and alternative donors. In unrelated CB transplantation, proximal ATG (from day −9 to −6) and rituximab are added in order to reduce primary graft rejection and post-transplant autoimmune cytopenia [122].

In LSD, a non-carrier well-matched unrelated donor is preferred to a carrier family donor. This is not so clear in the non-LSD, and so a carrier sibling sister might be used in X-ALD. In terms of stem cell source, CB is frequently preferred to BM since the post-HSCT chimerism has been reported to be higher and the interval between referral and transplant is likely the shortest (primary rejection might be higher using CB) [118]. BM rather than PB is usually preferred in family and unrelated adult donors. No ex vivo TCD is usually employed since this has been associated with graft loss [123].

While MPSIH is a standard indication for HSCT, in MLD, HSCT is usually reserved for late-onset variants of the disease, namely, juvenile and adult forms. For the early onset and rapidly progressing forms, GT has been shown to improve outcomes thanks to overexpression of the therapeutic enzyme systemically and locally in the CNS, resulting in prevention of progression of both motor and cognitive decline [124]. Based on these results, GT has been approved by EMA (Libmeldy®) and FDA (Lenmeldy®) for the treatment of pre-symptomatic Late Infantile and Early Juvenile (EJ) forms and early-symptomatic EJ forms.

In X-ALD, the best outcomes from HSCT are achieved when the transplant is undertaken early in the inflammatory disease and when there are early signs of demyelination on cerebral MRI (X-ALD Loes score < 9). A GT approach has been successful in X-ALD with the prevention of progression of cerebral inflammation in some boys, as after HSCT [125, 126]. GT is therefore not likely to be more effective than HSCT. Cases of myelodysplasia (in the range of 15% to 20%) associated with the integration of the lentivirus and its promoter (more powerful than that used in the MLD GT program) have been reported after GT in X-ALD [127], highlighting the importance of continuous and long-term monitoring for the risk of insertional mutagenesis. GT for X-ALD (Skysona®) is available in the USA for boys 4–17 years of age with early, active cerebral X-ALD.

Similar GT approaches are under clinical investigation for other LSD including MPSIH, MPSIIIA, MPSII with promising preliminary results. A phase III, multi-centre, randomized clinical trial is ongoing in Europe and the US to compare the safety and efficacy of GT versus HSCT in MPSIH. It is likely that GT will change the landscape of therapeutic approaches in some IEM in the coming years, together with the wide implementation of NBS strategies. Myeloablative conditioning is still required for engraftment of the genetically modified HSC. Some programs have included immune suppression with conditioning to prevent rejection of the genetically modified cells or the transgene product, but the exact role of such immune suppression will be refined in the coming years and with continuing investigations.

### Future perspectives

We anticipate that the number of allogeneic HSCTs performed for patients with inborn errors will continue to increase in both children and adults [128]. These guidelines, designed to ensure that patients receive care consistent with expert consensus and current best practices, will need regular updates as scientific understanding advances. We expect continued progress and new discoveries, particularly in areas such as personalized dosing of conditioning agents, less toxic antibody-based conditioning regimens, and improved strategies for patient selection. We are aware that many of the publications underlying these guidelines have been produced in centres in Europe and the US with few resource restrictions. Many of the recommendations made here, like timing of HSCT and dealing with comorbidities, will apply in centres all around the world. But clearly, one of the greatest challenges in improving HSCT outcomes for patients with IEI and IEM on a global scale will be to not only enable access to transplantation in countries with fewer resources, but also to adapt these recommendations to settings where some of the recommended agents or pharmacokinetic monitoring may not be accessible [129].
